# CDR2 and CDR2L line blot performance in PCA-1/anti-Yo paraneoplastic autoimmunity

**DOI:** 10.3389/fimmu.2023.1265797

**Published:** 2023-09-29

**Authors:** Nisa Vorasoot, Madeleine Scharf, Ramona Miske, Smathorn Thakolwiboon, Divyanshu Dubey, John R. Mills, Sean J. Pittock, Anastasia Zekeridou, Anthonina Ott, Andrew McKeon

**Affiliations:** ^1^Department of Laboratory Medicine and Pathology, Mayo Clinic, Rochester, MN, United States; ^2^Division of Neurology, Department of Medicine, Faculty of Medicine, Khon Kaen University, Khon Kaen, Thailand; ^3^The Institute for Experimental Immunology, Affiliated to Euroimmun AG, Lubeck, Germany; ^4^Department of Neurology, Mayo Clinic, Rochester, MN, United States

**Keywords:** autoimmune, ataxia, paraneoplastic, breast cancer, ovarian cancer

## Abstract

**Background:**

Purkinje cytoplasmic autoantibody type 1 (PCA-1)/anti-Yo autoimmunity is a common high-risk paraneoplastic neurological disorder, traditionally attributed antigenically to cerebellar degeneration–related protein 2 (CDR2), predominantly affecting women with gynecologic or breast adenocarcinoma. Single-modality CDR2 testing may produce false-positive results. We assessed the performance characteristics of the more recently purported major PCA-1/Yo antigen, CDR2-like (CDR2L), side by side with CDR2, in a line blot format.

**Methods:**

CDR2 and CDR2L were tested in six specimen groups (serum and cerebrospinal fluid (CSF)). Group 1, PCA-1/Yo mouse brain indirect immunofluorescence assay (IFA) positives; Group 2, PCA-1/Yo IFA mimics; Group 3, suspected CDR2 line blot false positives; Group 4, consecutive patient samples tested for neural antibodies over 1 year; Group 5, healthy subject serums; and Group 6, polyclonal (non-specific) immunoglobulin G (IgG)-positive serums.

**Results:**

Group 1: Of 64 samples tested, all but two were CDR2 positive (both CSF samples) and all were CDR2L positive. In individual patients, CDR2L values were always higher than CDR2. The two “CDR2L-only” positives were CSF samples with low titer PCA-1/Yo by IFA with serum negativity but with typical clinical phenotype. Group 2: All 51 PCA-1/Yo mimics were CDR2/CDR2L negative. Group 3: Nine samples [six of 1289 (0.47%) serums and three of 700 CSF samples (0.43%) were PCA-1/Yo IFA negative/CDR2 positive; two of the six available (serums from the same patient) were also CDR2L positive; the other four CDR2L negative had low CDR2 values (17–22). Group 4: Twenty-two patients had unexpected CDR2 or CDR2L positivity; none had tissue IFA positivity. Eleven of the 2,132 serum (0.5%) and three of the 677 CSF (0.4%) samples were CDR2 positive; median value was 19 (range, 11–48). Seven of the 2,132 serum (0.3%) and three of the 677 CSF (0.4%) samples were CDR2L positive; median value was 18 (range, 11–96). Group 5: All 151 healthy serum samples were negative. Group 6: One of the 46 polyclonal serum samples was CDR2L positive. Optimum overall performance was accomplished by requiring both CDR2 and CDR2L positivity in serum (sensitivity, 100%; and specificity, 99.9%) and positivity for CDR2L in CSF (sensitivity, 100%; and specificity, 99.6%).

**Conclusion:**

CDR2L provides additional PCA-1/anti-Yo sensitivity in CSF, and dual positivity with CDR2 provides additional specificity assurance in serum. Combining antigen-specific and tissue-based assays optimizes PCA-1/anti-Yo testing.

## Introduction

Purkinje cytoplasmic autoantibody type 1 (PCA-1, also known as anti-Yo) is a biomarker of paraneoplastic neurological autoimmunity, usually manifesting as cerebellar ataxia (paraneoplastic cerebellar degeneration) in women with gynecologic or breast adenocarcinoma (1, 2). The diagnosis is typically accomplished in serum or CSF by screening for a criterion-based pattern of patient antibody staining of neuronal cytoplasmic elements of rodent brain tissue by immunohistochemical assay [either indirect immunofluorescence assay (IFA) or immunoperoxidase-based] and confirmed by Yo antigen [cerebellar degeneration–related protein 2 (CDR2)]–specific immunoblot (3–5). CDR2 testing, when used in isolation or as a screening test, has association with significant numbers of false-positive results (6, 7). CDR2-like (CDR2L) is now recognized as another major antigen in PCA-1/Yo autoimmunity and, perhaps, the main antigen (8, 9). Testing utilizing CDR2L line blot or cell-based assay has been reported to have improved performance characteristics over CDR2 (6, 10). Here, we compared the performance of CDR2 and CDR2L antigens in a line blot format among six patient and control groups (among over 3,000 tested).

## Methods

### Standard protocol approvals, registrations, and patient consents

This retrospective study was approved by the Mayo Clinic Institutional Review Board (IRB, 21-001297). Medical records of patients who consented to research review were included.

### Sample groups tested

The following groups of patient samples were evaluated. Groups 1–4 were samples derived from clinical laboratory service, Neuroimmunology Laboratory, Mayo Clinic. Groups 5 and 6 were additional control samples tested. Neurological and cancer histories were obtained where possible for samples with unexpected results. **Group 1 (PCA-1/Yo IFA positives):** Samples (64) were serum (39) or CSF (25) from 48 patients identified in our clinical service laboratory (three were evaluated neurologically at Mayo Clinic, 34 elsewhere). All samples yielded PCA-1/Yo by tissue IFA (January 2019 to December 2022). Fifteen patients had serum/CSF pairs available for testing. **Group 2 (PCA-1/Yo IFA mimics):** There were 53 samples (30 serum and 23 CSF samples) with immunohistochemical staining mimicking but not fulfilling criteria for PCA-1/Yo (January 2019 to December 2022). **Group 3 (suspected CDR2 line blot false positives):** There were nine samples [six of the 1,289 serum (0.47%) and three of the 700 CSF (0.43%) samples] reflexed from IFA (27 February 2022 to 14 March 2023) without PCA-1/Yo by IFA, in which CDR2 was incidentally detected during confirmation testing for another antibody (e.g., anti-Hu). Six (five serum and one CSF samples) were available for CDR2L testing. **Group 4 (consecutive Mayo Clinic referred for neural antibody testing):** There were 2,809 consecutive available specimens (2,132 serum and 677 CSF samples; including 410 serum/CSF pairs), from patients all neurologically evaluated at Mayo Clinic, and referred for neural antibody testing in the Mayo Clinic Neuroimmunology Laboratory (19 September 2016 to 25 October 2017). **Group 5 (healthy subject serums):** There were 151 serum samples from healthy adult donors (EUROIMMUN). **Group 6 (a potential source of false positives through non-specific IgG interference):** There were 46 serum samples from patients without neurological disease but known to have polyclonal hypergammaglobulinemia (a potential interfering substance in IgG-binding assays).

### Serological testing

**Figure 1:**
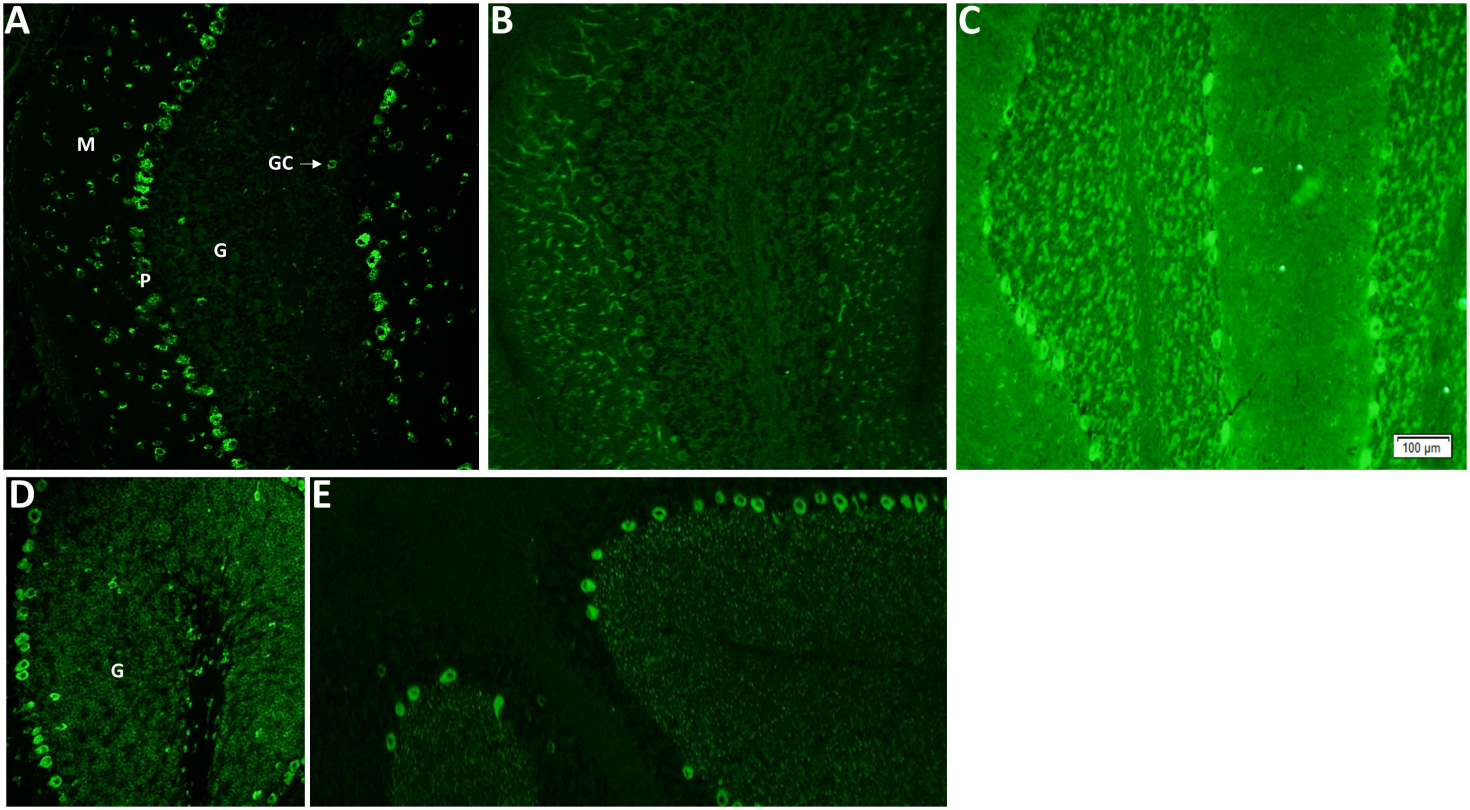
Indirect immunofluorescence assay utilizing mouse brain as substrate demonstrates Purkinje cytoplasmic antibody type 1 (PCA-1/Yo), other classified PCA antibodies, and mimics of PCA-1/Yo. **(A)** PCA-1-IgG/anti-Yo staining of cerebellum. Purkinje (P) neuron cytoplasm with a “chunky” appearance, scattered molecular (M) layer neurons and sparing of the granular (G) layer, save for isolated Golgi cells (GCs). Other classified PCA antibodies include PCA-2 (**B**; also known as MAP1B) and PCA-Tr (**E**; also known as DNER antibody). Unclassified mimics of PCA-1/Yo (protein target unknown) are diverse and exemplified in **(C**, **D)**; a potentially treacherous PCA-1/Yo mimic is signal recognition particle (SRP) antibody (a biomarker of necrotizing myopathy). Although it produces diffuse cytoplasmic staining, it is distinguishable by the presence of granular (G) layer staining, which is largely absent for PCA-1/Yo, and staining of non-neural elements (such as proximal gastrointestinal mucosa, not shown).

IFA utilizing a composite of murine brain, gut, and kidney was performed as previously described (11). Criteria for PCA-1/Yo by indirect murine tissue–based IFA include neuronally restricted cytoplasmic staining throughout the cerebrum, myenteric plexus, and cerebellum. The distinctive features of the cerebellar staining (**Figure 1A**) include Purkinje (P) neuron cytoplasm with a “chunky” appearance, scattered molecular (M) layer neurons, and sparing of the granular (G) layer, save for isolated Golgi cells (GCs). In addition, there should be an absence of staining of control tissues (such as gastrointestinal mucosa and kidney parenchyma). Examples of PCA-1 mimics are shown in **Figures 1B–E**. For CDR2 and CDR2L line blot, each patient specimen was incubated with multi-antigen–coated test strips (EUROIMMUN) and was tested as previously described (12). CDR2 and CDR2L purification had been achieved by immobilized metal affinity chromatography. Bound IgG was detected by application of enzyme-conjugated anti-human IgG catalyzing a color reaction, and densitometric data were acquired by EUROBlotOne/EUROLINEScan software (performed at Mayo Clinic in Groups 1–3 and 6; cutoff for positive was ≥15) or by flatbed scanner/EUROLINEScan software (performed at EUROIMMUN in Groups 4 and 5; cutoff for positive was ≥11).

Correlation of CDR2 and CDR2L values was evaluated using logistic regression analyses. Serum and CSF values were compared using Mann–Whitney U-test.

### Data availability

Anonymized data used for this study are available upon request.

## Results

### Group 1 (PCA-1/Yo IFA positives, 2019–2022)

All 48 patients were positive for PCA-1/Yo by tissue IFA (**Table 1**; **Figure 1A**). Median IFA titers were as follows: serum, 1:30,720 (range, 960–122,880; normal, ≤240); and CSF, 1:256 (range, 4–1,024; normal, ≤2). Typical of a PCA-1/Yo cohort, median age at testing was 64 years (range, 46–88 years), and all were women. The three patients neurologically assessed at Mayo Clinic had histories available. All had paraneoplastic cerebellar degeneration in the setting of adenocarcinoma (ovarian, 2; and breast, 1). Of 64 samples tested, all but two (96%) were CDR2 positive (median value of all positives, 132; range, 39–156), and all (100%) were CDR2L positive (median value, 186; range, 47–203). In serum, median value for CDR2 was 134 (range, 70–156) and that for CDR2L was 188 (range, 114–203). In CSF, median value for CDR2 was 127 (range, 5–148) and that for CDR2L was 178 (range, 47–195). Both CDR2L-only–positive samples were CSF (values, 47 and 115) and were from female patients, aged 50 and 64 years. Both were IFA serum negative (no CDR2/CDR2L testing possible as both serum samples had been discarded). Both had tissue IFA titers at the lower end of the positive range (end-point dilutions, 1:4 and 1:16). History, available in one of these two non-Mayo patients, revealed paraneoplastic cerebellar degeneration in the setting of breast adenocarcinoma.

**Table 1:** Demographic, clinical, and serological data for Groups 1, 2, 3, 5, and 6.

CDR2/CDR2L line blot tested groups	IFA	CDR2+	CDR2L+	Notable neurological histories	Cancer
**1. PCA-1/Yo IFA positive**	PCA-1/Yo, 64 *Serum titers* 1:30,720 (range, 960–122,880; normal, ≤240) *CSF titers* 1:256 (range, 4–1,024; normal, ≤2)	62/64 *Serum values* *39/39 (134, 70–156)* *CSF values* *24/26 (127, 39–148)*	64/64 *Serum values* *39/39 (188, 114–203)* *CSF values* *26/26 (178, 47–192)*	*Mayo patients* F/46. Cerebellar ataxia F/54. Cerebellar ataxia F/62. Cerebellar ataxia *CDR2-negative cases* F/50. Cerebellar ataxia F.64. NA	Ovarian adenocarcinoma Ovarian adenocarcinoma Breast adenocarcinoma Breast adenocarcinoma NA
**2. PCA-1/Yo mimics**	PCA-1/Yo mimics, 53	0	0	–	–
**3. Other samples reflexed to line blot from IFA**	Positive serums Negative, serum Negative, serum Negative, serum ANNA-1, serum ANNA-1, serum Positive CSFs Negative, CSF	6/1,289* * Serum values* *17* *17* *22* 44** 59** 3/700*** * CSF value* *19*	2/5 CDR2+ serums * Serum values* * Neg* *Neg* * Neg* *148* *155* 0/1 CDR2+ CSFs * CSF value* *Neg*	M/79. Peripheral neuropathy F/67. Peripheral neuropathy F/91. NA F/81. Cerebellar ataxia F/81. Cerebellar ataxia NA	None None NA Small cell carcinoma Small cell carcinoma NA
**5. Normal healthy serum controls**	0/151	0/151	0/151	–	–
**6. Polyclonal hypergammaglobulinemia controls (without neurological disease)**	0/1	0/46 *Neg*	1/46 (118) *Serum value* *118*	–	–

*Five of the six CDR2-positive serum samples were available for CDR2L testing. **Duplicate serums from same patient. ***One of the three CDR2-positive CSF samples were available for CDR2L testing. ANNA-1, antineuronal nuclear antibody type 1; F, female; M, male; NA, not available; PCA-1, Purkinje cytoplasmic antibody type 1 (also known as anti-Yo); Neg, negative.

### Group 2 (PCA-1/Yo IFA mimics, 2019–2022)

All 53 samples were CDR2 and CDR2L negative (**Table 1**).

**Figure 2:**
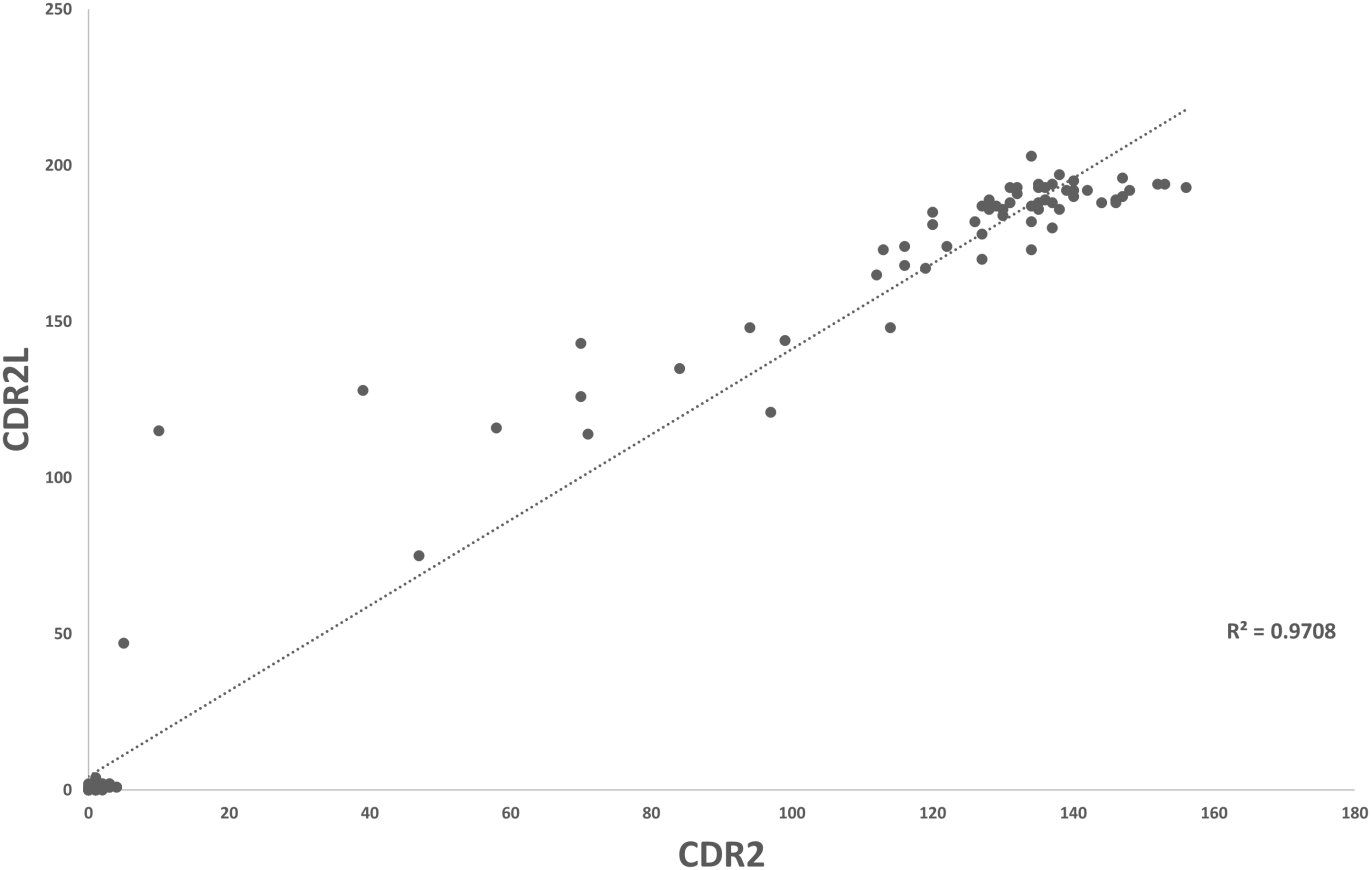
Scatter plot demonstrating correlation of CDR2- and CDR2L-positive values in patients with true-positive PCA-1/Yo (Group 1) and mimics (Group 2).

Overall, for data for Groups 1 and 2, there was a good correlation between CDR2 and CDR2L values (R^2^ = 0.970) (**Figure 2**). CDR2L values were always higher than CDR2 values in individual patients. CDR2L values (but not CDR2 values) were significantly higher in serum than that in CSF (p = 0.037).

### Group 3 (suspected CDR2 line blot false positives, 2022–2023)

The historic CDR2 values for the six positive serum samples ranged from 17 to 59 (median, 22) and the three CSF samples from 19 to 30 (median value, 21). Six of the samples were available for CDR2L testing (five serum and one CSF samples). The solitary CDR2-positive CSF specimen tested was CDR2L negative. Two of the five PCA-1 IFA-negative/CDR2-positive serums were also CDR2L positive (value, 148 and 155, both from the same patient) (**Table 1**). This patient had antineuronal nuclear antibody type 1 (ANNA-1) detected by IFA and was also confirmed ANNA-1 (anti-Hu) antibody positive by line blot. Other small cell carcinoma paraneoplastic antibodies [SRY-box transcription factor 1 (SOX-1) (119) and Zic family member 4 (ZIC4) (145)] had also been detected by line blot only during clinical evaluation. The patient was an 80-year-old woman who presented with cerebellar ataxia and had small cell lung carcinoma (no history of breast or gynecologic adenocarcinoma). IFA for the three remaining specimens from two women and one man was reflexed from IFA for initially suspected non–PCA-1/Yo paraneoplastic antibodies (amphiphysin, 2; and ANNA-1, 1), and all ultimately were determined to be negative for those other antibodies. CDR2 values in those three samples were close to the cutoff for positive (≥15; range, 17–22), and CDR2L was negative in all. None of the three had CSF available for testing.

### Group 4 (consecutive Mayo Clinic patients referred for neural antibody testing 2016–2017)

For context, two Mayo patients had expected PCA-1/Yo autoimmunity during the 1-year epoch (both had serum tested and one had CSF tested, and all samples positive). Both had paraneoplastic disorders in the setting of adenocarcinoma (of the breast and fallopian tube, respectively). Neither had samples residual for CDR2L testing. An additional 22 patients had unexpected CDR2 or CDR2L positivity, where tissue IFA was PCA-1/Yo negative. Thirteen patients were CDR2-only positive, eight patients were CDR2L-only positiv, and one patient was positive for both CDR2 and CDR2L (**Table 2**). For CDR2, 11 of the 2,132 serum (0.5%) and three of the 677 CSF (0.4%) samples were positive; median value was 19 (range, 11–48). For CDR2L, seven of the 2,132 serum (0.3%) and three of the 677 CSF (0.4%) samples were positive; median value was 18 (range, 11–96). Just one serum/CSF pair was weakly positive (**Table 2**, Patient 20). Eighteen of the 22 patients (82%) were men, and none had typical anti-Yo neurological or oncological phenotypes. For 21 of the 22 patients, positivity was for one analyte only: 13 were CDR2-only positive (11 were positive in serum and two in CSF) or eight were CDR2L-only positive (six were positive in serum, one in CSF, and one weakly positive in serum and CSF); all but one (Patient 13) had non-autoimmune neurological diagnoses (encephalitis), and just one (Patient 14) had active cancer (melanoma). The remaining patients (12) who had dual-CDR2/CDR2L positivity in CSF (serum negative) had a steroid-responsive presumed autoimmune meningoencephalitis.

**Table 2:** Demographic, clinical, and serological data for Group 4 patients positive for CDR2 or CD2L by line blot.

Pt	Sex	Age	Sample type(s)	Tissue IFA	CDR2	CDR2L	Neurological disorder	Neoplasia
1	M	68	S	Neg	43	Neg	Dizzy spells	None
2	M	46	S	Neg	11	Neg	Neuropathy	None
3	M	68	S C	Neg	12 Neg	Neg Neg	Motor neuron disease	MGUS
4	M	71	S	Neg	13	Neg	Neuropathy	Prostate adenocarcinoma, history
5	M	50	S	Neg	16	Neg	Ataxia	None
6	M	44	S	Neg	30	Neg	Motor neuron disease	None
7	F	48	S	Neg	24	Neg	MSA	None
8	F	34	S	Neg	22	Neg	Ataxia	None
9	M	54	S	Neg	14	Neg	Neuropathy	None
10	M	41	S	Neg	34	Neg	Neuropathy	None
11	M	75	S	Neg	13	Neg	None	None
12	M	60	S C	Neg Neg	Neg 48	Neg 96	Meningoencephalitis, steroid responsive	None
13	M	74	S C	Neg Neg	Neg 17	Neg Neg	Encephalitis	SQCC skin, history
14	M	51	C	Neg	22	Neg	Neuropathy	Metastatic melanoma
15	M	55	S	Neg	Neg	31	Paresthesias NOS	None
16	M	49	S	Neg	Neg	18	Cognitive symptoms	None
17	M	56	S	Neg	Neg	12	Cognitive difficulties ans seizures	None
18	M	69	S	Neg	Neg	52	PSP	None
119	M	67	S	Neg	Neg	18	Neuropathy	Prostate adenocarcinoma, history
20	M	66	S C	Neg	Neg Neg	11 15	Motor neuron disease	None
21	F	52	S	Neg	Neg	13	Myelopathy	None
22	M	5	C	Neg	Neg	30	Congenital malformation, seizures	None

Adenoca, adenocarcinoma; MGUS, monoclonal gammopathy of undetermined significance; MSA, multiple-system atrophy; NOS, not otherwise specified; PSP, progressive supranuclear palsy; SQCC, squamous cell carcinoma; Neg, negative.

### Group 5 (healthy subject serums)

None of the 151 serum samples were CDR2 or CDR2L positive (**Table 1**).

### Group 6 (potential source of false positives through interference)

One of the 46 polyclonal serum samples (2%) was CDR2L positive (value, 118) (**Table 1**). This sample was then tested on IFA and was negative. None of the 46 samples were CDR2 positive.

To assess sensitivity and specificity, we aggregated all the data from patient groups where both CDR2 and CDR2L had been evaluated equally (Groups 1, 2, 4, 5, and 6) and used the presence or absence of tissue IFA criteria as the gold standard for PCA-1 autoimmunity. We did not include Group 3 data because CDR2L testing was only performed on the available nine false-positive CDR2-positive cases from that cohort of 1,989 samples tested. Sensitivity data were as follows: CDR2 in serum, 100%; CDR2L in serum, 100%; CDR2 in CSF, 92%; and CDR2L in CSF, 100%. Specificity data were as follows: CDR2 in serum, 99.5%; CDR2L in serum, 99.7%; CDR2 in CSF 99.6%; and CDR2L in CSF, 99.6%. Combining CDR2 and CDR2L with a requirement for detection of both analytes above the cutoff to yield a positive result, sensitivity was 100% in serum and 92% in CSF, and specificity was 99.9% for both specimen types. Combining CDR2 and CDR2L with a requirement for one analyte above the cutoff to yield a positive result, sensitivity was 100% in both serum and CSF samples, and specificity was 99.2% for serum and 99.6% for CSF.

## Discussion

PCA-1 (anti-Yo) autoimmunity is the second most common high-risk paraneoplastic antibody after ANNA-1 (anti-Hu) detected in clinical laboratory practice (13). Although PCA-1/Yo can be readily identified by tissue-based assay, there are also mimics encountered in laboratory practice that closely resemble the PCA-1/Yo staining pattern, and, thus, a robust confirmatory test is critically important. CDR2-based molecular testing has been the main source of confirmatory testing. However, questions have been raised regarding the utility of CDR2 because of the potential for false positives and false negatives (6). In addition, CDR2L has been proposed as the main antigen in PCA-1/Yo autoimmunity, which prompted us to study these antigens across a number of patient groups encompassing known PCA-1/Yo autoimmunity and potential sources of false positives (8, 9).

We addressed sensitivity of the line blot by comparing CDR2 and CDR2L as reflex confirmatory tests among samples meeting IFA criteria for PCA-1/Yo. The available histories from patients assessed neurologically at the Mayo Clinic indicated that this group was representative of PCA-1/Yo autoimmunity (women, with cerebellar ataxia and breast or gynecologic cancer). Both antigens had equivalent sensitivity to confirm PCA-1/Yo IFA results in serum (100%). However, CDR2L values were universally higher than CDR2 in PCA-1/Yo IFA-positive cases. This finding is consistent with those from a study using a peptide-based phage-immunoprecipitation platform (14). In particular, we found that CDR2L was more sensitive than CDR2 in CSF, being positive in two (of 17) low-titer IFA-positive cases where CDR2 was negative. Serum testing was also negative in those two cases. Thus, sensitivity for PCA-1/Yo autoimmunity is optimized by testing in CSF where serum is negative and confirming by CDR2L.

We addressed the specificity of the line blot by testing for CDR2 and CDR2L among samples with IFA mimicking PCA-1/Yo, sequential Mayo Clinic patients in whom neurological autoimmune testing had been requested, and healthy control serums and serums with the potential to produce interference in an immunoassay (polyclonal IgGs). Although the non-detection of CDR2/CDR2L among samples producing IFA features mimicking PCA-1/Yo and healthy controls was reassuring, some false positives were encountered for both analytes among consecutive Mayo Clinic patient samples generally referred for neurological autoimmune testing. These false positives were more commonly detected in serum than in CSF, usually for one analyte only and occurred more frequently for CDR2 than CDR2L. Overall, requiring positivity for both CDR2 and CDR2L optimized specificity for both serum and CSF samples, but there was some loss of sensitivity for CSF. Thus, optimum overall performance might be accomplished by requiring positivity for both CDR2 and CDR2L in serum and positivity for either in CSF. In the Mayo Clinic experience to date, the values for CDR2 false-positive results (highest being 59 for serum and 19 for CSF) have been lower than that for true positives (lowest being 70 for serum and 39 for CSF). It should be borne in mind that some laboratories screen by line blot and reflex to tissue-based assay for confirmation, the opposite of the approach described here.

The two patients encountered with immune-mediated neurological disorders in whom both CDR2 and CDR2L were detected at intermediate or high values, but without PCA-1/Yo observed by tissue IFA, did not have PCA-1/Yo typical phenotypes. The immunological and clinical significance of these findings are unknown. One patient was a man with steroid-responsive meningoencephalitis without cancer with dual positivity in CSF but not in serum. The other was a woman with cerebellar degeneration but in the context of ANNA-1 (anti-Hu) by tissue IFA, with dual-CDR2/CDR2L positivity in serum (CSF not available) and had the ANNA-1–associated neoplasm (small cell carcinoma) rather than PCA-1/Yo–associated breast or gynecologic neoplasia. Consistent with the immune response against multiple neural antigens encountered in small cell carcinoma, she was also positive for Hu, SOX-1, and ZIC4 by line blot (15, 16). It is possible that the PCA-1/Yo staining pattern by IFA was masked by coexisting ANNA-1 staining. The coexistence of PCA-1/Yo and ANNA-1 antibodies in patients with dual cancers is extremely rarely reported (17).

Limitations of our study include missing clinical data for some subjects, and the focus on line blots currently used commonly in clinical practice. Future studies will need to examine promising CDR2L cell–based assays in a similarly high-throughput laboratory practice (10). In addition, our sensitivity assessment was undertaken using PCA-1/Yo IFA criteria as the gold standard rather than clinical cohorts. However, in our experience, classical sensitivity assessments of paraneoplastic antibodies are uninformative because of the rarity of the individual antibodies. Even among well-defined seropositive autoimmune ataxias PCA-1/Yo accounts for just 30% of patients (18). PCA-1/Yo was recently reported to account for 1.6% of all positives in an academic clinical service laboratory (19). Clinical specificity was addressed among the very large and clinically diverse Group 4 cohort (consecutive Mayo Clinic patients tested over 13 months).

We conclude that, for PCA-1/Yo autoimmunity, CDR2L in the line blot format improves sensitivity in CSF and is optimized for specificity in serum when combined with CDR2. Our observations give emphasis to the importance of pairing tissue-based and antigen-specific assays for the diagnosis of paraneoplastic neurological disorders (3). Although a specificity of >99% can seem reassuring, in high-throughput clinical laboratory practice, even a small loss of specificity can translate into a false positives “issue” (20). Ideally, testing laboratories should screen for PCA-1/Yo by tissue-based assay and confirm by antigen-specific assay, such as the line blots described here. Line blot data used in isolation should be interpreted with caution, particularly for low positive results in serum. Pairing CDR2 and CDR2L may help overcome some interpretative issues.

## Data availability statement

The raw data supporting the conclusions of this article will be made available by the authors, without undue reservation.

## Ethics statement

The studies involving humans were approved by Mayo Clinic Institutional Review Board. The studies were conducted in accordance with the local legislation and institutional requirements. The participants provided their written informed consent to participate in this study.

## Author contributions

NV: Data curation, Investigation, Methodology, Writing – review & editing. MS: Conceptualization, Data curation, Formal Analysis, Investigation, Methodology, Validation, Writing – review & editing. RM: Conceptualization, Data curation, Formal Analysis, Investigation, Methodology, Validation, Writing – review & editing. ST: Formal Analysis, Writing – review & editing. DD: Data curation, Formal Analysis, Writing – review & editing. JM: Data curation, Formal Analysis, Writing – review & editing. SP: Data curation, Formal Analysis, Writing – review & editing. AZ: Data curation, Formal Analysis, Writing – review & editing. AO: Data curation, Formal Analysis, Investigation, Methodology, Validation, Writing – review & editing. AM: Conceptualization, Data curation, Formal Analysis, Funding acquisition, Investigation, Methodology, Supervision, Validation, Writing – original draft, Writing – review & editing.
